# Extensive Workup on Early-Onset Fibromyalgia: A Case Report

**DOI:** 10.7759/cureus.21714

**Published:** 2022-01-29

**Authors:** Guy Treves, Mustaqueem Qazi

**Affiliations:** 1 Medicine, St. George's University School of Medicine, True Blue, GRD; 2 Internal Medicine, Desert Oasis Healthcare, Indio, USA

**Keywords:** unusual presentation, autoimmune pots, autoimmune, early onset fibromyalgia, fibromyalgia

## Abstract

This case report details a unique case of fibromyalgia manifested at a very early age, and the patient’s suffering as a result of mis- and under-diagnosis of her condition for over a decade. For most of her life, the patient has had seemingly unrelated bouts of pain, neurological conditions, and psychiatric disturbances, and has seen numerous specialists for these conditions to no avail. The lengthy history discussion and physical examination findings, detailed in this report, revealed a progressive disease course. Providing the patient with drafts of the compiled history and data has encouraged her to reseek specialist care. With this revealed history, physical examination information, and lab results, progress has been made to diagnose and treat the patient’s numerous conditions. In addition to the unique fibromyalgia presentation, the case report provides compelling justification for physicians to consider broad differential diagnoses, engage in patient-centered discussion about treatment progression, and to strengthen and maintain the patient-provider relationship.

## Introduction

Fibromyalgia is a chronic condition that causes exaggerated pain sensations and is prevalent in up to 6.6% of the general female population [1]. It typically presents at middle age but can occur in teen years [2]. Patients with the condition typically suffer from a host of comorbidities that can greatly vary in severity [3]. Since many patients experience only a subset of symptoms, fibromyalgia might only be diagnosed years after symptom onset, or remain undiagnosed entirely, while each symptom is treated on an isolated basis.

This report presents an extensive workup for an early-onset case of fibromyalgia on an unusually young female patient who suffered from bouts of pain, neurological conditions, and psychiatric disturbances for the majority of her life with largely unsuccessful treatment. At the time of her initial assessment, she had become convinced that the medical system would be unable to remedy her ailments. The purpose of this workup was to attempt to rule out underlying causes, make appropriate referral recommendations, and provide the patient a sense of reassurance after feeling hopeless for years. The timeline given refers to the age of the patient and to the days before and after the initial encounter. The patient was diagnosed with fibromyalgia at the age of 19 years.

This study was done by a medical student under the supervision of a physician. It is not a legal document and does not assign a diagnosis to the patient nor does it prescribe a treatment plan.

## Case presentation

History of presenting illness

In early childhood, as early as five years old, the patient has been sensitive to light, noise, and touch. Since elementary school, she has had difficulties with balance and coordination movements, such as catching objects, tripping while walking, and playing sports. The patient’s right wrist started hurting with a constant feeling of soreness at around 12 years of age and was treated with a brace. The left wrist started hurting with bouts of soreness at around 13 years of age and was treated with a brace as well. The patient’s wrist pain worsened for two years before visiting a doctor for this issue. At this time, a wrist ultrasound, an x-ray, and an MRI of the arms were normal. A sports medicine specialist placed the patient on piroxicam which did not relieve the pain and had side effects including loss of appetite and nausea. 

At the age of 15 years,** **the patient was referred to occupational therapy for 14 weeks for wrist strengthening exercises. This resulted in shooting pains up the thumb and lateral arm bilaterally. Custom wrist and thumb braces were then used 24/7 for seven weeks and did not improve the pain or the original wrist soreness. At the age of 16 years,** **a nerve conduction study and electromyogram was was conducted and found to be normal. A rheumatologist was consulted for the first time and assessed that the patient’s condition is likely not rheumatologic. Physical therapy was prescribed with nerve glides, neck rubs, and wrist strengthening exercises. The physical therapy did relieve the shooting pain up the arms but not the wrist pain. New-onset back pain began at this time and was also worked on in physical therapy with little improvement. At this point, the patient felt dread and that none of her providers were useful and bore this condition independently for some time. At this time she had to stop most of her physically active hobbies due to intolerable bouts of pain. 

At the age of 17 years,** **the patient consulted her primary care provider, who suspected based on the previous rheumatologic assessment that the patient’s condition is likely not due to fibromyalgia. She was referred to an orthopedic surgeon who recommended a regimen of stationary bike and swimming exercises, the use of topical creams and cannabidiol oil, and referred the patient to physical therapy again. An x-ray showed mild degeneration at the L5 level. Physical therapy provided the patient with a transcutaneous electrical nerve stimulation unit and a heating pad, both of which were useful in relieving some of the pain in her feet and back. The patient also consulted a chronic pain provider and received CT-guided steroid injections in the lower back. She experienced severe pain with difficulty walking for three days after the injection, and after those three days, the back pain resumed. She refused additional injection treatment. She was then referred to a pain psychiatrist who failed to diagnose her condition and did not initiate treatment. She was then referred to acupuncture but discontinued treatment as the needles were too painful.

The patient had an emergency room admission for a severe episode of back pain. Norco and cyclobenzaprine relieved this pain. Two weeks afterward there was another emergency room admission for a severe episode of back pain during which prescribed ibuprofen did not relieve the symptoms. At this time, a consulting spine surgeon could not discern the cause for her pain and ordered an MRI with the following impression - minimal synovial fluid/effusions at bilateral L3/L4 and L4/L5 facet joints, likely representing mechanical stress. No other spinal abnormalities were noted. An aberrant right subclavian artery was found incidentally. 

At the age of 18 years, the patient was diagnosed with fibromyalgia by a second rheumatologist. Positive antinuclear antibodies were found and noted to have unclear etiology with no clinical manifestation of systemic lupus erythematosus. A regimen of daily stretches was recommended which became progressively painful. There was no improvement in the pain.

At the age of 19 years, a third rheumatologist ordered extensive lab tests and x-rays of feet, hands, neck, knees, and lower limbs. Lab results were notable for lupus anticoagulant, antinuclear antibodies, anti-thyroid peroxidase antibodies, and anti-carbonic anhydrase antibodies (Table 1). The patient was unable to obtain the x-ray results.

**Table 1:** Laboratory values *Abnormal values. SGU: specific gravity units; EU: endotoxin units

Comprehensive metabolic panel		Reference
Glucose	80	65-99 mg/dL
Blood urea nitrogen (BUN)	12	7-20 mg/dL
Creatinine	0.73	0.50-1.00 mg/dL
Estimated glomerular filtration rate non-African American	119	> or = 60 mL/min/1.73m^2^
Estimated glomerular filtration rate African American	138	> or = 60 mL/min/1.73m^2^
BUN/creatinine ratio	Not applicable	6-22 (calculated)
Sodium	138	135-146 mmol/L
Potassium	4.2	3.8-5.1 mmol/L
Chloride	102	98-110 mmol/L
Carbon dioxide	19 L*	20-32 mmol/L
Calcium	9.8	8.9-10.4 mg/dL
Total protein	7.5	6.3-8.2 g/dL
Albumin	4.5	3.6-5.1 g/dL
Globulin	3.0	2.0-3.8 g/dL (calculated)
Albumin/globulin ratio	1.5	1.0-2.5 (calc)
Total bilirubin	0.6	0.2-1.1 mg/dL
Alkaline phosphatase	56	36-128 U/L
Aspartate aminotransferase	18	12-32 U/L
Alanine aminotransferase	18	5-32 U/L
Erythrocyte sedimentation rate by modified Westergren	17	< or = 20 mm/h
Aldolase	3.7	< or = 8.1 U/L
Vitamin B12	312	200-1100 pg/mL
Folate	572	>280 ng/mL red blood cells
Total creatine kinase	77	29-143 U/L
Total vitamin D 1,25 (OH)2	68	18-72 pg/mL
Vitamin D3, 1,25 (OH)2	68	18-72 pg/mL
Vitamin D2, 1,25 (OH)2	<8	18-72 pg/mL
Thrombin clotting time	16	13-19 seconds
Complete blood count		
White blood cell count	8.3	3.8-10.8 thousand/uL
Red blood cell count	4.37	3.80-5.10 million/uL
Hemoglobin	13.7	11.7-15.5 g/dL
Hematocrit	40.0	35.0-45.0%
Mean corpuscular volume	91.5	80.0-100.0 fL
Mean corpuscular hemoglobin	31.4	27.0-33.0 pg
Mean corpuscular hemoglobin concentration	34.3	32.0-36.0 g/dL
Red cell distribution width	12.0	11.0-15.0%
Platelet count	376	140-400 thousand/uL
Mean platelet volume	9.9	7.5-12.5 fL
Absolute neutrophils	5279	1500-7800 cells/uL
Absolute lymphocytes	2357	850-3900 cells/uL
Absolute monocytes	515	200-950 cells/uL
Absolute eosinophils	100	15-500 cells/uL
Absolute basophils	50	0-200 cells/uL
Absolute nucleated red blood cells	0	0 cells/uL
Neutrophils	63.6	%
Lymphocytes	28.4	%
Monocytes	6.2	%
Eosinophils	1.2	%
Basophils	0.6	%
Antibodies titer and pattern		
Human leukocyte antigen B27	Negative	Negative
Antinuclear antibodies screen with immunofluorescence assay	Positive*	Negative
Antinuclear antibodies titer	1:80 H*	40 titer
Antinuclear antibodies pattern	Nuclear, homogeneous*	
Thyroglobulin antibodies	<1	< or = 1 IU/mL
Complement component C3C	133	83-193 mg/dL
Complement component C4C	38	15-57 mg/dL
Total complement (CH50)	>60 H*	31-60 U/mL
Rheumatoid factor	<14	<14 IU/mL
C-reactive protein	2.0	<8.0 mg/L
SCL-70 antibody	<1.0 negative	<1.0 negative
Thyroid peroxidase antibodies	96 H*	<9 IU/mL
Thyroid-stimulating hormone antibody	Negative	Negative
Sjogren's antibody (SS-A)	<1.0 negative	<1.0 negative
Sjogren's antibody (SS-B)	<1.0 negative	<1.0 negative
Cyclic citrullinated peptide antibodies (IgG)	<16	19 units
Centromere B antibody	<1.0 negative	<1.0 negative
Ribonucleoprotein antibody	<1.0 negative	<1.0 negative
Anti-Smith antibody	<1.0 negative	<1.0 negative
Total hepatitis B core antibody	Non-reactive	Non-reactive
Hepatitis B core antibody (IgM)	Non-reactive	Non-reactive
Hepatitis C antibody	Non-reactive	Non-reactive
Lupus anticoagulant	Detected*	Not detected
Partial thromboplastin time - lupus anticoagulant screen	48 H*	< or = 40 seconds
Dilute Russell viper venom time screen	46 H*	< or = 45 seconds
Hexagonal phase confirm	Weakly positive*	Negative
Dilute Russell viper venom time confirm	Positive*	Negative
1:1 mix dilute Russell viper venom time	Corrected	Corrected
DNA double-stranded antibodies by Crithidia luciliae (IFA)	Negative	Negative
Salivary protein (SP 1) IgG antibodies	4.0	<19.9 EU/mL
Salivary protein (SP 1) IgA antibodies	<1.0	<19.9 EU/mL
Salivary protein (SP 1) IgM antibodies	<1.0	<19.9 EU/mL
B2 glycoprotein IgG antibodies	<9	≤ 20 SGU
B2 glycoprotein IgM antibodies	<9	≤ 20 SGU
B2 glycoprotein IgA antibodies	<9	≤ 20 SGU
Carbonic anhydrase VI (CA VI) IgG antibodies	21.6 H*	<19.9 EU/mL
Carbonic anhydrase VI (CA VI) IgA antibodies	1.4	<19.9 EU/mL
Carbonic anhydrase VI (CA VI) IgM antibodies	<1.0	<19.9 EU/mL
Parotid specific protein (PSP) IgG antibodies	2.4	<19.9 EU/mL
Parotid specific protein (PSP) IgA antibodies	<1.0	<19.9 EU/mL
Parotid specific protein (PSP) IgM antibodies	<1.0	<19.9 EU/mL
14.3.3η (eta) protein	<0.2	<0.2 ng/mL
Cardiolipin antibody (IgA)	<11	IgA isotype
Cardiolipin antibody (IgG)	<14	IgG isotype
Cardiolipin antibody (IgM)	<12	IgM isotype

Current encounter

At the time of evaluation, the patient complains of allodynia and diffuse pain upon contact throughout the body. She can tolerate her weighted blanket and lightly brush her hair. She eventually shaved her head because of the pain. She occasionally has shooting pain down all of her limbs, occurring at least every couple of days and often several times per day. This pain differs in intensity from 4-5 out of 10 to 9-10 out of 10 and lasts anywhere from a few seconds to an hour. The patient further complains of restless legs, having to continuously get up through the day, and often waking up at night to walk around the house for no apparent reason. A list of medications and allergies can be referenced below (Table 2).

**Table 2:** Medications and allergies

Current medications	Side effects
Seasonique	-
Cymbalta 30mg twice daily	-
Gabapentin 300mg thrice daily, weaning	Sleepiness, loss of appetite
Magnesium 250mg twice daily	-
Fish oil	-
Vitamin D 35 mcg daily	-
Zofran as needed for nausea	-
Previous medications	
Lexapro	Did not do enough for depression, anxiety, obsessive-compulsive disorder
Piroxicam	Appetite loss, nausea
Enskyce	Severe reaction including intense vomiting leading to emergency room visit
LoSeasonique	No effect
Norco	Prescribed at emergency room, very helpful with pain
Flexeril	Prescribed at emergency room, effective relaxant but causes excessive tiredness
Meclizine	Extreme dizziness
Zoloft	Switched to Cymbalta due to nerve pain
Allergies	
Sensitive skin to soaps and chlorine	Can tolerate gentle soaps
Bee allergy/sensitivity	Patient has an EpiPen but never used
Piroxicam	Nausea and loss of appetite
Enskyce	Severe reaction including intense vomiting

Psychiatric history

The patient started feeling depressed at five years of age and has had numerous depressive episodes since. She started seeing a psychiatrist for worsening mental health at the age of 16 years. She was diagnosed with depression, generalized anxiety disorder, dermatillomania, and mild-to-moderate obsessive-compulsive disorder. She was prescribed escitalopram, which did not improve any of the symptoms. Sertraline had a positive impact on all her disorders. She was switched to duloxetine because of its therapeutic effects on nerve pain and it is working well at this time. She is looking to get cognitive behavior therapy for dermatillomania and her chronic pain. The patient also complains of night sweats, which began just after starting the psychiatric medications. She often wakes up drenched in sweat and shivering regardless of the room temperature.

Head, eyes, ear, nose, and throat history

Migraines and Headache Episodes

Episodes of headache began at the age of 15 years. They occurred frequently in high school and since improved to occur about once a month. An episode starts with sharp pain in the back of the head. There is a tight-band sensation occurring on one half of the head and can switch multiple sides during a single episode. It is associated with an aura described as focusing and unfocusing vision, increasing sound sensitivity, and nausea. Sitting in a dark, quiet room typically relieves these symptoms. The patient was told to take a 500mg magnesium supplement, which has improved the symptoms.

Dizziness

The dizziness, begun at the age of 16 years, is associated with seeing all-black starting at the center of vision and spreading out, a loss of balance, and occasionally a spinning room sensation. The dizziness waxes and wanes through the day. An episode is worsened by closing the eyes. An episode can be triggered by looking up, lying on the back, and sudden head movements. Sometimes the episodes occur at random. An EKG at the time of the dizziness onset was normal. The given diagnosis was overactive vagal nerve and the patient was reassured that she would grow out of it by her 20s and for now should treat it by consuming electrolytes. At the age of 18 years, the dizziness worsened. At that time, the patient was prescribed gabapentin and titrated up to 900mg. This led to the loss of appetite, nausea, and worsening dizziness, and the patient is currently in the process of weaning off the gabapentin.

Additional Head, Eyes, Ear, Nose, and Throat Symptoms

The additional head, eyes, ear, nose, and throat symptoms include the following: (1) temporomandibular joint -** **pain in the temporomandibular joint began at the age of 16 years and waxes and wanes in severity. There is a clicking sound on the left temporomandibular joint and pain on the right temporomandibular joint whenever the mouth is closed forcefully or from a wide opening such as in yawning and chewing. (2) Lymphadenopathy -** **the patient complains of a nodule behind both ears starting at the age of 18 years. It has not subsided. It grows and reduces and is assumed to be a lymph node. It is painful when it is at the largest size. The nodules enlarge and reduce independently of each other. At the time of the encounter, the right nodule is enlarging and the left is reducing. (3) Moles - two moles present on the scalp are painful when touched. One was removed at the age of 18 years. A third growing mole was removed from the left labia majora at the age of 10 years. (4) Sinuses -** **the patient has equalization problems with her sinuses. She cannot scuba dive, feels pain while on airplanes and driving through mountains, and has had great difficulty equalizing the pressures. (5) Hyperacusis -** **the patient says her hearing is very sensitive and can hear the buzzing from light fixtures. She often has to sit in dark rooms or turn off fluorescent lights as they are too loud to tolerate. There is episodic ringing in the ears that improves with head position changes. (6)Mouth -** **there is taste sensitivity to spices including black pepper and mint. Any portion of the tongue or gums hurt on contact. There has been one episode of strep throat in high school. (7) Miscellaneous - there are no changes to vision outside of the dizziness and migraine episodes, no loss of smell, no loss of taste, no enlarged lymph nodes in the neck or jaw, and no diagnoses of thyroid disorders.

Gastrointestinal history

In high school, the patient had painful hiccup episodes for 20-30 minutes following eating. The hiccups were loud, deep, and spaced anywhere from a few seconds to 30 seconds. There was no sensation of stuck food in the throat. The frequency of these episodes has reduced and presently they occur about twice a month. There has never been diarrhea or constipation.

Gynecologic history

The patient's gynecologic history includes the following: (1) breast mass -** **the patient found a small, painful mass in her left breast at the age of 16 years. This was diagnosed as mastitis and disappeared with a 20-day antibiotic course. A right breast mass occurred at the age of 17 years and disappeared with two weeks of antibiotics. There have not been any masses since. There has never been nipple discharge. (2) Menstruation - menarche started at the age of 14 years. By the age of 15 years, the patient was experiencing monthly menstruation with seven to nine days of bleeding, along with pain several days before, during, and after. Oral birth control was started at the age of 16 years due to menstrual pain. Menstruation, controlled with Seasonique, now occurs once every three months and lasts four days with pain and light bleeding. (3) Vaginal pain -** **the patient has severe pain with insertion of a tampon or other devices. The pain began as an uncomfortable sensation when trying to insert tampons at the onset of menstruation with discomfort the whole time the tampons were in. The pain has worsened to the point that insertion of even thin tampons is impossible. The patient also attempted to use a menstrual cup but could never insert the cup. This pain is always present and is not associated with menstruation. An abdominopelvic ultrasound at the age of 19 years was normal and transvaginal ultrasound was deferred due to pain. She will be consulting a gynecologist shortly after this encounter. (4) Vaginal discharge - the patient has never been sexually active. Discharge has been present since the onset of menstruation. The discharge is yellowish-white, sometimes liquid and sometimes clumpy. The discharge is continuous and has never remitted. The patient was put on a seven-day course of antibiotics for this discharge four months ago which did not stop the discharge nor her constant pain. There have been two asymptomatic urinary tract infections but no burning urination.

Family history and review of systems

The patient's father has rheumatoid arthritis treated with methotrexate. Her mother has generalized anxiety. There is a family history of Hashimoto’s thyroiditis. Both parents have had an Ashkenazi Jewish panel prior to conception which was negative. There are no breathing difficulties, coughs, fever, or racing heart outside of anxiety episodes. The patient has to get up once or twice per night to urinate. She cannot dehydrate herself before bedtime as she feels lightheaded.

Physical examination findings

**Figure 1:**
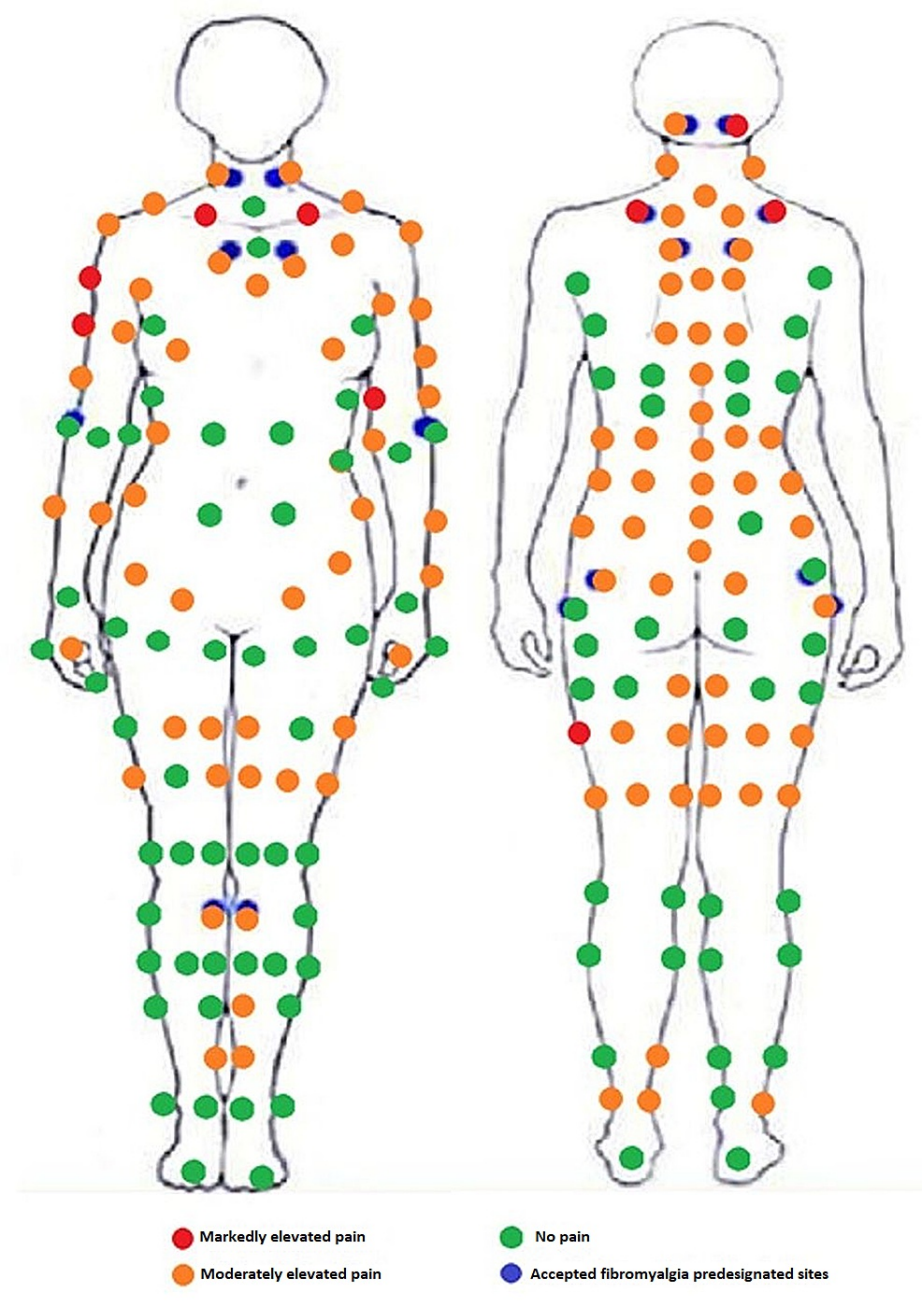
Diagrammatic representation of pain felt upon palpation

The patient's physical examination findings include the following: (1) gynecological - there is a scar on the left labia, presumed from previous mole removal. Discharge is present in the form of small white clumps without odor. There is no tenderness along the vestibule. The bimanual examination is deferred due to the patient’s fear of pain. (2) Breast - there are no visible skin abnormalities. There is a palpable, smaller than 1cm, mobile mass with regular borders at the lower, outer areola border bilaterally. Pressing on the nipples elicits pain. (3) Abdomen - there are no hernias nor bruits. Bowel sounds are present. There is no tenderness to palpation. The liver is not palpable. (4) Cardiovascular - there is no elevated jugular venous pressure. There are no thrills or heaves. The point of maximal impulse is at the fifth intercostal space on the left. There are normal S1 -S2 sounds. There are no S3 or S4 sounds, murmurs, rubs, or gallops. (5) Respiratory - there is a symmetric rise and fall of the chest. There is no tenderness on palpation. Vesicular breath sounds are heard in all lung quadrants. (6) Head, eyes, ears, nose, and throat - the patient can elicit the temporomandibular joint popping noises by widely opening and closing her mouth as described in the history. There is a surgical scar and lack of hair to the right of the central hair whorl, presumed to be the mole extraction site. There is an 8mm mole left of the central hair whorl on the scalp that is painful to palpation. There is a palpable nodule behind each ear. The right nodule is larger than the left. Palpating the temporomandibular joint does not elicit pain. The thyroid is nonpalpable. (7) Neurologic - the pattern of excessive pain elicited upon light palpation is described in Figure 1. Cranial nerve examination is within normal limits. Ophthalmologic examination is normal. Weber’s test does not lateralize. Rinne’s test shows air conduction greater than bone conduction bilaterally however bone conduction can be heard for a remarkable time (more than 30 seconds) while the subsequent air conduction can be heard for an additional minute. Muscle bulk is appropriate on all limbs and torso. Power is 5/5 in all extremities. Brachial, biceps, triceps, and knee reflexes are +2 bilaterally. All digits can discriminate two points 3mm apart. All toes can discriminate two points 6mm apart. There are symmetric and appropriate pain, vibration, proprioception, and graphesthesia sensations. Romberg’s test is positive. Heel-to-toe walking, tiptoe walking, and heel walking are all normal. Rapid alternating movement, finger-to-nose, and heel-to-shin tests are normal.

## Discussion

The patient was advised of her conditions and the collated results. Emphasis was placed on validating the patient’s decade-long pain, and reassuring her that her symptoms were now being holistically considered to the fullest possible extent. Her plan of care is to be focused on ruling out possible underlying conditions and referring the patient for pertinent specialist evaluation.

She was advised the following: an FM/a test could be useful in diagnosing or ruling out fibromyalgia [4]. A complete autoimmune antibody panel and endocrinology studies should be performed to rule out conditions such as thyroid, pituitary, and adrenal disorders, myasthenia gravis, and celiac disease. Vitamin studies can also be performed to rule out other causes of neuropathy [5]. If all of these investigations prove to be benign, it may be appropriate to consult with a geneticist for a whole-genome sequencing study including mitochondrial disorders [6] and Ashkenazi Jewish-related conditions [7]. Specialist providers should also be consulted. A neurologist should be consulted for imaging and to rule out multiple sclerosis, tumors, or other neurological causes. Gynecologic care should be re-established to attempt to regulate menstruation and the accompanying pain, with further evaluation should pharmacologic treatment fail. The collated history and, once acquired, the lab investigations, should be forwarded to rheumatology for re-evaluation.

Follow-up investigations

After the encounter, the patient was referred to several specialists and for extensive laboratory investigations for continued evaluation. The patient has completed labs and an autoimmune panel (Table 1). The follow-up investigations include (1) primary care provider -** **general labs are unremarkable but there are positive autoimmune markers. The patient should proceed with referrals. (2) Gynecology - the patient was assessed as having dysmenorrhea and breakthrough bleeding on birth control pills. She was advised to stop taking birth control after four years of failed efforts to control her menstrual cycles and pelvic pain and started on Low-Ogestrel once daily with no placebo. At any time the patient may elect to undergo diagnostic laparoscopy to further investigate the cause of pelvic pain. (3) Rheumatology - the elevated antinuclear antibodies could be a possible early indication of rheumatologic disease due to the family history. Further, it is believed that the lupus anticoagulant is a false-positive. The patient was advised that her problem is likely not rheumatologic in nature and was referred to an endocrinologist for further evaluation. (4) Neurology - the patient was diagnosed with migraines with auras and tension headaches, and there are no neurologic red flags to warrant imaging at this time. She will be prescribed rizatriptan to be taken as needed. The patient noted that her smartwatch heartbeat monitor jumps 40 points when she stands. A tilt table test confirmed postural orthostatic tachycardia syndrome (POTS) and is a possible explanation for the patient’s exercise intolerance and extensive fatigue. Differential diagnoses include benign paroxysmal positional vertigo and obstructive sleep apnea. A sleep study was prescribed for the latter. The patient will be referred to cardiology for further POTS evaluation. 

Analysis

It is difficult to clearly delineate a chronological chain of events, stemming from the patient’s signs and symptoms. Given the insidious history and physical examination findings, this patient displays many manifestations of fibromyalgia according to the American College of Rheumatology 1990 guidelines [8]. The patient displays widespread out-of-proportion pain and greater than mild tenderness at 14 of the 18 fibromyalgia designated sites. This is further supported by a nonfocal neurological examination with the exception of hyperacusis. This patient suffers from many of the comorbidities associated with fibromyalgia including depression and anxiety, restless leg syndrome, elevated autoimmune titers, migraines, and possible vulvodynia or vaginismus. A differential diagnosis, or perhaps comorbidity, is POTS. The patient has several manifestations of POTS including chronic fatigue, heightened pain, recurrent headaches, a history of unexplained hiccups, and a positive tilt table test. This condition has a number of proposed etiologies including autonomic neuropathy, endocrine dysregulation, chronic hypovolemia, and cardiovascular deconditioning [9]. The positive autoimmune titers and the history of restless legs and night sweats hint at autonomic dysregulation, and offer possible etiologies in this patient. While the patient could be suffering from fibromyalgia, POTS, or both, the etiology of either remains unclear. Even though several interfering factors can cause a false-positive lupus anticoagulant and require confirmatory testing, the other elevated titers of autoimmune antibodies and increased complement levels may point to lupus, Hashimoto's thyroiditis, or other developing rheumatological diseases as the true cause of the patient's ailments [10]. A normal complement level offers less support for lupus or may indicate a subclinical disease process [11]. A complete complement analysis including the markers of complement activation and routine monitoring of thyroid function, coagulability, and erythrocyte sedimentation rate may provide further insight into a progressive condition. Furthermore, it is unclear whether any of the aforementioned disease processes explain the gynecologic symptoms. While it is possible that the pelvic pain arises from a neurogenic source, differential diagnoses include endometriosis, ovarian cysts, and failure of the hypothalamic-pituitary-ovarian axis. Thus, vaginal pain could be a linked symptom or separate comorbidity. The vaginal discharge may be an unremitting yeast infection, possibly candidiasis, which could have an etiology linked to the autoimmune process. Given the unconventional nature of these symptoms, it is possible that the morbidities have an unexplored cause. Further testing and repeating laboratory studies will be essential to rule out possible causes and gather more data.

## Conclusions

This patient has faced an insidious and prolonged disease course, compounded by a decade-long challenge to receive a clear diagnosis and appropriate medical treatment. She has supported the creation of this report out of desperation and a desire to have her medical challenges affirmed and validated, with no expectation that any actual treatment will follow. At this time, progress had been made in aiding this patient through her challenge. Continuous follow-up with the patient including specialist recommendation, lab result interpretation, and reassurance has positively improved her future outlook and mental mindset. While there is a set plan for continued care, any additional course of action, opinions, and all suggestions that may benefit the patient are welcomed and may be sent to the author.

Two key learnings may be taken from this report. First, seemingly unrelated signs and symptoms can manifest as a result of an underlying condition. While it is often easier to focus on the treatment of a single symptom at a time, repeated failures or prolonged treatment should prompt expansion of the differential diagnoses to rarer or more improbable syndromes. Second, maintaining the patient-provider relationship, in itself, can be a source of encouragement and hope for the patient. Showing that the patient’s complaints are valued and considered, that there is mutual understanding, and, if necessary, that treatment options beyond the specialty of the provider are discussed, may prevent the patient from entirely abandoning the medical system.
